# Detecting the fractal physical activity pattern in aged adults with cerebral small vessel disease

**DOI:** 10.3389/fnagi.2025.1569582

**Published:** 2025-04-28

**Authors:** Hóngyi Zhào, Wei Wei, Fang Lv, Jie Shen, Yonghua Huang

**Affiliations:** ^1^Department of Neurology, Seventh Medical Centre of PLA General Hospital, Beijing, China; ^2^Department of Psychiatry, No 984 Hospital of PLA, Beijing, China; ^3^Department of Gerontology, No 984 Hospital of PLA, Beijing, China

**Keywords:** actigraphy, aging, small vessel disease, neurodegenerative disease, wearable

## Abstract

**Background:**

Actigraphy is widely used to detect a decline in physical activity in aged individuals with cerebral small vessel disease (cSVD). Disturbed fractal physical activity has been reported in aged adults with Alzheimer’s disease (AD), mild cognitive impairment (MCI), and other neuropsychiatric disorders.

**Objectives:**

To analyze the fractal physical activity pattern in elderly patients with cSVD.

**Methods:**

From May 2021 to August 2023, 55 patients with cSVD aged 60–80 years admitted to the seventh medical center of PLA General Hospital were included. The presence of lacunes, white matter hyperintensities, cerebral microbleeds, and perivascular spaces on magnetic resonance images (MRI) were rated independently. Furthermore, these MRI markers were summed in a score of 0–4, representing all cSVD features combined. Detrended fluctuation analysis (DFA) was used to evaluate the fractal physical activity fluctuations at multiple time scales. The relationship between the fractal physical activity pattern and physical activity and sleep quality was analyzed with partial Pearson correlation analysis.

**Results:**

Individuals with a low severity cSVD burden showed a significant tendency toward a random fractal pattern relative to those with a higher severity cSVD burden. Similar results were obtained when comparing the lacune positive and negative groups. In aged adults with cSVD, fractal disturbance was associated with an average level of physical activity and not sleep quality.

**Conclusion:**

These findings demonstrate the presence of obvious fractal physical activity complexity in aged adults with cSVD.

## 1 Introduction

Due to the rapid advances in technology of wearable devices used for assessing daily activities, actigraphy is now frequently used to examine physical activity in clinical and research studies involving patients with neurodegenerative diseases (Boroojerdi et al., 2019; Saif et al., 2020). Actigraphic devices are used to quantify many clinical features related to physical activity, such as energy expenditure, circadian rhythm, and apathy severity, enabling unobtrusive monitoring with a reduced dependency on the human observer (Cai et al., 2022; Vandenbussche et al., 2024; Zhao et al., 2023). It was recently revealed that the self-similarity of actigraphy-derived motor activity fluctuations, when magnified across different time scales, known as fractal motor activity regulation, is linked to Alzheimer’s disease (AD) and mild cognitive impairment (MCI) (Li et al., 2018; Huber et al., 2019).

In addition to the linear analysis of physical activity levels, researchers have focused on the scale-invariant or “fractal” properties of locomotor activity, characterized by similar temporal structures across different time scales (Raichlen et al., 2019). This kind of fractal stability is found in many physiological variables, including heart rate, respiration, gait, brain activity, etc. (Pittman-Polletta et al., 2013). Fractal regulation of motor activity is believed to reflect system integrity and adaptability (i.e., the ability to respond to external changes while maintaining certain stability for orchestrated internal physiological functions) (Knapen et al., 2021). Furthermore, changes in fractal physical activity patterns have been demonstrated to be better predictors of morbidity and mortality than classical biomarkers (Lin et al., 2010; Joustra et al., 2018; Lin et al., 2021).

Cerebral small vessel disease (cSVD) represents a common condition in older adults, occurring in approximately 80% of the general population over the age of 60 years (Moran et al., 2012; Siejka et al., 2018). However, the early detection of cSVD is difficult in clinical practice (Zhao et al., 2024). Our previous studies demonstrated that cSVD severity was associated with the physical activity level in aged adults, as measured with both subjective assessment questions and wearable sensors (Cai et al., 2022; Zhào et al., 2020); however, there is still a lack of reports regarding motor activity fractal fluctuations in aged individuals with cSVD. Thus, the purpose of the current study was to investigate the fractal physical activity patterns in elderly patients with cSVD and to determine the relationship between fractal disruption and cSVD magnetic resonance imaging (MRI) markers, such as white matter hyperintensities (WMHs), lacunes, cerebral microbleeds (CMBs), and perivascular spaces (PVS).

## 2 Materials and methods

### 2.1 Participants

This clinical cross-sectional observational study included 55 elderly patients with cSVD from the Department of Neurology at the Seventh Medical Center of PLA General Hospital from May 2021 to August 2023. The Academic Ethic Committee of the Biological Sciences Division of the Seventh Medical Center of PLA General Hospital approved the study.

The exclusion criteria included the following: presence of major stroke, other causes of leukoencephalopathy (e.g., immune, demyelination, genetic), major psychiatric disorders (diagnosed with the Diagnostic and Statistical Manual of Mental Disorders [DSM-IV]), use of psychotropic medications or drugs with the side effect of risk of falling (e.g., tranquilizers/sedatives, diuretics, antiparkinsonian drugs), MRI contraindications, dementia (diagnosed with ICD-10) or a mini-mental state examination score < 23 points (Ma et al., 2022), sleep abnormalities, recency of surgery which could impact nighttime activity, recency of surgery impacting daytime mobility, and the use of walking aids.

### 2.2 MRI measurements

A 3.0 T MRI scan (Discovery MR750; GE Healthcare, Waukesha, WI, USA) of the brain showed white matter lesions that were compatible with SVD grade 1, 2, or 3. The brain imaging protocol, based on a slice thickness of 5 mm and an interslice thickness of 1.5 mm, employed the following parameters: for T1 fluid-attenuated inversion recovery images, the repetition time (TR) was 1,750 ms, echo time (TE) was 23 ms, TI was 780 ms, and field of view (FOV) was 24 cm; for T2-weighted images, TR was 7,498 ms, TE was 105 ms, and FOV was 24 cm.

### 2.3 Total cSVD burden score

The total cSVD burden score was calculated according to our previous protocol (Zhao et al., 2022; Xia et al., 2023). One point was allocated to each of the following MRI parameters: moderate-to-severe WMHs (Fazekas score: 2–3), presence of lacunes, CMBs (Wardlaw et al., 2013), and moderate-to-severe basal ganglia–perivascular spaces (PVS) (semi-quantitative rating > 1), with total scores ranging from 1 to 4 points (Staals et al., 2014; details were shown in Supplementary Figure 1). To elucidate whether actigraphic data differed according to the cSVD burden score, subjects were also divided by the total cSVD burden score. To compare the parameters in detail, we also grouped individuals binarily into the WMH positive, WMH negative; lacunes positive, lacunes negative; PVS positive, PVS negative; and CMB positive, CMB negative groups, according to our previous study (Xie et al., 2024).

### 2.4 Wearable actigraphy

In accordance with our protocol, after the MRI scan, all cases were asked to wear the ActiGraph GT3X+ device (ActiGraph, Pensacola, United States) on the non-dominant wrist all day (except when swimming or bathing) for 7 days.

ActiLife software (ActiGraph, Pensacola, FL, USA) was used to download data after each wear period. Data with sufficient wear time were chosen and analyzed.

This work obtained actigraphic data at the epoch of 60 s using the corresponding software. Due to the triaxial accelerometer nature of the ActiGraph GT3X+ device, this work sampled vector magnitude (VM) using the formula below:


$$\mathrm{VM}=\sqrt{X^{2}+Y^{2}+Z^{2}}$$

where X, Y, and Z are VM counts in the X-, Y-, and Z-axes, respectively.

Bedtime and wake time from the sleep diary were used to define sleep–wake variables. The sleep quality variables consisted of sleep efficiency (SE), total sleep time (TST), times of awakenings (TA), and average duration of awakenings (ADA) (Zhao et al., 2022).

### 2.5 Fractal analysis

**FIGURE 1:**
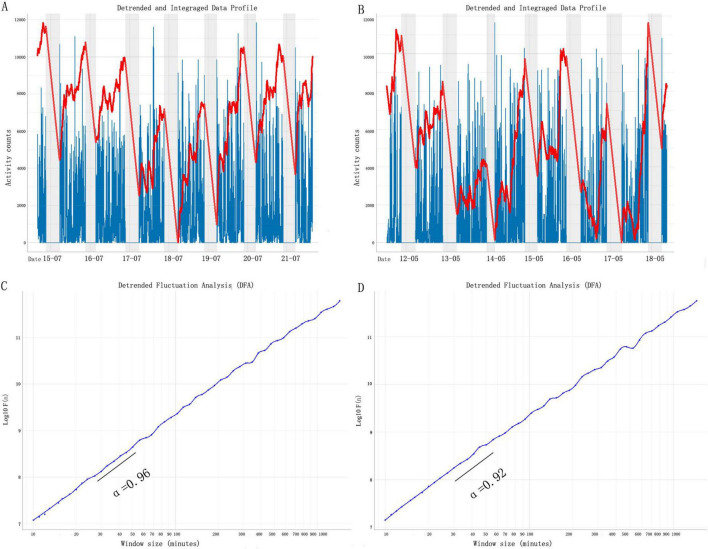
Fractal pattern of daily motor activity fluctuations. Representative motor activity recordings of two participants. The participant (cSVD burden score = 0) whose activity is shown in (A), has a DFA α = 0.96 **(C)**; the participant (cSVD burden score = 2) whose activity is shown in panel **(B)**, has a DFA α = 0.92 **(D)**. The gray shaded area indicates 9 p.m. to 7 a.m. DFA results of the signals in panels **(A,B)**. cSVD, cerebral small vessel disease; DFA, detrended fluctuation analysis.

Detrended fluctuation analysis (DFA) was performed on each subject using the nonlinear T series software PUZHEN Rhythm. This method was described in detail elsewhere (Raichlen et al., 2019) (see Figure 1). Briefly, in a DFA, time-series data are first integrated, following the removal of the global mean of the signal. Next, the time-series is divided into a series of non-overlapping windows of some size, n. Finally, the root mean square of residuals from a least-squares regression of the time-series within each window (detrending) is calculated to compute the fluctuation amplitude, F(n). These steps are then repeated across a series of 25 window sizes ranging from 10 min to 7 h in length. The fractal physical activity complexity of the time-series is determined by the slope, α, of the least-squares regression line relating F(n) to window size (t). If α = 0.5, the time-series does not include long-range correlations in fluctuations, suggesting fluctuations are mainly white noise. If 0.5 < α ≤ 1.0, the time-series contains positive long-range correlations in fluctuations, indicating decreasing fractal randomness as α nears 1.0. If α > 1.0, the time-series begins to be more regular or less responsive to external changes as α approaches 1.5. Thus, in analyses of motor activity data, α-values distant from 1.0 are considered representative of unhealthy fractal physiological pattern disruption.

In humans, aging and dementia lead to a breakdown in the multiscale pattern in motor activity that can be characterized by different correlations (indicated by different α) over two distinct time scale regions with the boundary at ∼1.5–2 h (Gao et al., 2021). Thus, two scaling exponents of F(n) were calculated in this study: α1 at <90 min and α2 from 2 h up to 10 h (Figure 1). Note that the transitional region of time scales between 1.5 and 2 h was omitted.

### 2.6 Statistical analysis

The Mann-Whitney U test was performed to assess continuous nonparametric variables. The Student’s *t*-test was used to analyze continuous parametric variables. Partial Pearson correlation analysis was performed to calculate the correlation between the α and VM and sleep–wake variables, which was controlled for age, sex, and educational level. A *P*-value < 0.05 was statistically significant. All statistical analyses were conducted using a statistical software package (SPSS, version 25.0; IBM Corp., Armonk, NY, USA).

## 3 Results

Figure 1 shows the demographic characteristics of all individuals. Sex (male: 35.71% vs.70.00% vs. 54.55% vs. 65%; *P* = 0.283), height (163.21 ± 7.51 cm vs. 165.80 ± 6.89 cm vs. 170.00 ± 6.74 cm vs. 166.95 ± 7.96 cm; *P* = 0.453), weight (66.86 ± 11.39 kg vs. 68.60 ± 12.83 kg vs. 69.91 ± 7.96 kg vs. 68.25 ± 15.55 kg; *P* = 0.074), and VM counts (1721.10 ± 4.26 vs. 710.29 ± 112 vs. 828.32 ± 384.40 vs. 710.82 ± 339.75; *P* = 0.320) did not reach statistical significance. Individuals with a low cSVD burden score (1 point) were found to be younger (60.43 ± 9.20 years) than those with a high cSVD burden score (2–4 points) (70.60 ± 6.13 years vs. 65.45 ± 11.39 years vs. 73.35 ± 8.76 years; *P* = 0.001).

Patients with a low cSVD burden score (1 point) exhibited a higher α relative to those with a low cSVD burden (2 points) (0.97 ± 0.04 vs. 0.92 ± 0.04 vs. 0.92 ± 0.04 vs. 0.92 ± 0.04; *P* = 0.011). Whereas, for α1, α2, and Δα, we did not find obvious differences among groups (details are shown in Table 1). All sleep quality variables, including SE, TST, TA, and ADA, showed no statistically significant difference between groups. Details are shown in Supplementary Table 1.

**TABLE 1:** Demographic characteristics and Hurst exponent of participants.

Item	cSVD burden score				*P*-value
	**1**	**2**	**3**	**4**	
**Demographic characteristics**	***N* = 14**	***N* = 10**	***N* = 11**	***N* = 20**	
Men, %	5 (35.71)	7 (70.00)	6 (54.55)	13 (65.00)	0.283
Age, mean (SD), years	60.43 (9.20)	70.60 (6.13)	65.45 (11.39)	73.35 (8.76)	0.001*^#^^&^
Height, mean (SD), cm	163.21 (7.51)	165.80 (6.89)	170.00 (6.74)	166.95 (7.96)	0.163
Weight, mean (SD), kg	66.86 (11.39)	68.60 (12.83)	69.91 (7.96)	68.25 (15.55)	0.951
VM, mean (SD), counts	1721.10 (3210.59)	710.29 (112.00)	828.32 (384.04)	710.82 (339.75)	0.032
α	0.97 (0.04)	0.92 (0.04)	0.92 (0.04)	0.93 (0.04)	0.011*^#^^&^
α1	0.95 (0.08)	0.96 (0.05)	0.92 (0.06)	0.93 (0.04)	0.576
α2	0.99 (0.10)	0.93 (0.12)	0.93 (0.09)	0.92 (0.08)	0.235
△α	−0.03 (0.15)	0.03 (0.14)	−0.00 (0.11)	−0.03 (0.21)	0.818

cSVD, cerebral small vessel disease; SD, standard deviation; VM, vector magnitude.

*P < 0.05 cSVD burden score = 1 vs. cSVD burden score = 2.

^#^*P* < 0.05 cSVD burden score = 1 vs. cSVD burden score = 3.

^&^*P* < 0.05 cSVD burden score = 1 vs. cSVD burden score = 4.

Lacunes-negative subjects were shown a higher α relative to lacunes-positive individuals (0.95 ± 0.05 vs. 0.92 ± 0.04; *P* = 0.007) (details are shown in Table 2); however, no statistical differences in fractal physical activity complexity were observed between individuals grouped by other MRI biomarkers (WMH, PVS, and CMBs) (data are shown in Supplementary Tables 2–4).

**TABLE 2:** Hurst exponent of participants divided according to lacunes.

Item	Lacunes negative (*N* = 20)	Lacunes positive (*N* = 35)	*P*-value
α	0.92 (0.04)	0.96 (0.05)	0.007**
α1	0.93 (0.06)	0.96 (0.07)	0.016*
α2	0.97 (0.10)	0.93 (0.09)	0.142
△α	−0.01 (0.16)	−0.02 (0.18)	0.878

*P < 0.05 between groups.

**P < 0.01 between groups.

Furthermore, correlation analyses were adopted to analyze the relationship between fractal physical activity complexity, VM counts, and sleep quality parameters. We found that both α (*r* = 0.564; *P* < 0.001) and α2 (*r* = 0.414; *P* = 0.008) showed a significant association with WM counts. On the contrary, neither α1 (*r* = 0.008, *P* = 0.957) nor Δα (*r* = −0.269; *P* = 0.062) were significantly associated with VM counts. There was no marked relationship between fractality and sleep quality. Details are shown in Table 3.

**TABLE 3:** Partial Pearson correlation between Hurst exponent and locomotor activity as well as sleep quality.

	α		α1		α2	
	***r*-value**	***P*-value**	***r*-value**	***P*-value**	***r*-value**	***P*-value**
VM	0.564	0.000***	0.008	0.957	0.414	0.003**
SE	−0.001	0.995	0.099	0.538	−0.105	0.560
TST	0.020	0.913	−0.005	0.980	0.054	0.766
TA	−0.037	0.836	−0.081	0.654	0.057	0.751
ADA	0.008	0.967	−0.235	0.188	0.070	0.699

Adjustment for age, sex, height and weight. ADA, average duration of awakenings; SE, sleep efficient; TA, times of awakenings; TST, total sleep time; VM, vector magnitude.

**P < 0.01.

***P < 0.001.

## 4 Discussion

There were two main findings in the current cross-sectional study. First, aged individuals with cSVD showed a significant reduction in fractal motor activity complexity, with a trend of randomness reflected by a reduced α-value. Second, in aged patients with cSVD, the fractal physical activity pattern was closely correlated with VM counts, a parameter reflecting physical activity. Previous studies have implied that irregular retinal microcirculation and electroencephalography fractality could reflect subclinical pathological changes in cerebral vascular disease (Al-Qazzaz et al., 2018; Gao et al., 2023). In 2022, another group of researchers reported that D_f_(w), an index measuring the complexity of a self-similar and irregular structure of Willis circle and its tributaries, was a promising, non-invasive vascular neuroimaging marker for asymptomatic cSVD with WMH (Aminuddin et al., 2022). When combined with our actigraphy results, these findings indicate that disrupted fractal fluctuation occurs in multiple dimensions of brain impairment. As we know, identifying and quantifying self-similarity from both topographic dimension (lung, brain, and cerebral vascular) and temporal dimension (actigraphy, electrocardiogram and electroencephalography) is a challenge (Pittman-Polletta et al., 2013). From a comprehensive perspective, we inferred that cSVD needed to be investigated with more systematic approaches in future.

Based on actigraphic devices, it is evidenced that fractal physical activity complexity is broken down into many types of neuropsychiatric disorders. For example, Gao et al. (2021) reported an obvious degradation in fractal regulation in elderly patients with AD (α ≈ 0.85). Another study discovered that depressed patients showed an altered fractal pattern (α ≈ 1.20) toward a smoother organization. It seemed that neither a decreased α (toward randomness, e.g., AD) nor an increased α (toward order, e.g., depression) reflected the physiological systems in healthy adults. The present study showed a more random fractal fluctuation in patients with cSVD (α ≈ 0.92) relative to aged controls (α ≈ 0.96). This finding supports a recently published report, which found that fractal dysregulation (reduced α) was associated with gross chronic infarcts, chronic microinfarcts, and arteriolosclerosis (Li et al., 2024). To take it further, we attempted to identify the fractal pattern among groups with different neuroimaging markers. The results demonstrated a diminished fractal complexity in aged patients with lacunes; however, no other statistical differences in fractality were found when we compared groups according to WMH, PVS, and CMB. To our knowledge, this is the first report to assess the fractal complexity in aged patients with cSVD.

The loss or reduction of fractal fluctuations indicated a physiological control system which was less complex, less adaptive to perturbations (Pittman-Polletta et al., 2013). Reduced α-value discovered in the present study could be an indicator for the increased vulnerability for other brain lesions in cSVD patients. For example, it has been evidenced that patients with isolated cerebellar infarction, WMH rather than infarct volume and topography, determined clinical outcomes, such as vertigo, nausea, unsteadiness, and limb ataxia (Grips et al., 2005). Besides, high overall cSVD burden was associated with increased risks of unfavorable outcomes in patients with large vessel occlusion stroke receiving endovascular treatment (Cheng et al., 2024).

We intended to determine the relationship between fractal physical activity complexity, average level of physical activity (VM counts), and sleep quality. In community-dwelling older adults, the fractal physical activity pattern was reported to be associated with levels of moderate-to-vigorous physical activity (Raichlen et al., 2019). Similarly, a positive correlation between fractal physical activity complexity and VM counts was found in the present study. The suprachiasmatic nuclei of the hypothalamus (SCN) have been found to contribute to fractal physical activity regulation (Pittman-Polletta et al., 2013). Together with our previous results demonstrating the abnormal VM counts and circadian rest–activity rhythm in patients with cSVD (Cai et al., 2022; Zhao et al., 2023), we inferred that cSVD (characterized by global arteriolosclerosis and lacunar infarcts) could impact the SCN and internal clock. Furthermore, it has been reported that the α-value in smaller and larger time scales differed, with α1 linked to mood and α2 linked to circadian dysfunction (Li et al., 2019). We revealed a relationship between α2 and VM counts. As has been mentioned by Hu et al. (2013), α2 could predict changes in neurotensinergic neurons, which are major circadian neurotransmitters in the SCN (Li et al., 2019). Thus, it is reasonable to suggest that neurotensin is related to modulating physical activity (Ramirez-Virella and Leinninger, 2021; Ivanov et al., 2007).

Similar to our previous work (Zhào et al., 2025), we did not find a relationship between the fractal physical activity pattern and sleep quality in patients with cSVD. This might be explained by the fact that the impact of some factors (e.g., sleep apnea) on sleep quality could not be assessed directly by actigraphy (Zuurbier et al., 2015).

Several shortcomings of this study need to be considered. First, the sample size was small. Second, this study has a cross-sectional design, and thus, the causality of the relationship between cSVD and disturbed fractal physical activity regulation could not be concluded. Third, there was a lack of control group. In future study, we would collect more participants and encompass aged-matched elderly without cSVD.

In summary, based on MRI imaging, all these findings imply a negative influence of cSVD severity on the SCN and fractal neurophysiological control in aged adults.

## Data Availability

The original contributions presented in this study are included in this article/Supplementary material, further inquiries can be directed to the corresponding author.

## Supplementary material

Supplementary Figure 1 Total small-vessel disease score features and categories.
