# Supplementary material for: Detecting the fractal physical activity pattern in aged adults with cerebral small vessel disease
Source: Front Aging Neurosci. 2025 Apr 28;17:1569582. doi: 10.3389/fnagi.2025.1569582 (PMC12066675; doi:10.3389/fnagi.2025.1569582)
Supplement: Supplementary file 1 [file Data_Sheet_1.docx]

**Supplementary Table 1 Sleep quality of participants**

| Item | cSVD burden score | | | | P value |
| --- | --- | --- | --- | --- | --- |
|  | 1 | 2 | 3 | 4 |  |
| Demographic characteristics | N=14 | N=10 | N=11 | N=20 |  |
| SE, mean (SD), % | 5(35.71) | 7(70.00) | 6(54.55) | 13(65.00) | 0.283 |
| TST, mean (SD), minutes | 60.43(9.20) | 70.60(6.13) | 65.45(11.39) | 73.35(8.76) | 0.014*^#^ |
| TA, mean (SD), times | 163.21(7.51) | 165.80(6.89) | 170.00(6.74) | 166.95(7.96) | 0.402 |
| ADA, mean (SD), minutes | 66.86(11.39) | 68.60(12.83) | 69.91(7.96) | 68.25(15.55) | 0.125 |

ADA, average duration of awakenings; cSVD, cerebral small vessel disease; SD, standard deviation; TA, times of awakenings; TST, total sleep time.
