# Supplementary material for: Detecting the fractal physical activity pattern in aged adults with cerebral small vessel disease
Source: Front Aging Neurosci. 2025 Apr 28;17:1569582. doi: 10.3389/fnagi.2025.1569582 (PMC12066675; doi:10.3389/fnagi.2025.1569582)
Supplement: Supplementary file 2 [file Data_Sheet_2.docx]

**Supplementary Table 2 Hurst exponent of participants divided according to WMH**

| Item | Fazekas score | | | P value |
| --- | --- | --- | --- | --- |
|  | 1 | 2 | 3 |  |
| Demographic characteristics | N=25 | N=18 | N=12 |  |
| α | 0.94(0.05) | 0.94(0.04) | 0.92(0.05) | 0.205 |
| α1 | 0.95(0.07) | 0.95(0.06) | 0.92(0.07) | 0.336 |
| α2 | 0.96(0.11) | 0.94(0.09) | 0.90(0.07) | 0.168 |
| △α | -0.02(0.16) | 0.01(0.11) | -0.05(0.25) | 0.691 |

WMH: white matter hyperintensities
