# Supplementary material for: Detecting the fractal physical activity pattern in aged adults with cerebral small vessel disease
Source: Front Aging Neurosci. 2025 Apr 28;17:1569582. doi: 10.3389/fnagi.2025.1569582 (PMC12066675; doi:10.3389/fnagi.2025.1569582)
Supplement: Supplementary file 3 [file Data_Sheet_3.docx]

**Supplementary Table3 Hurst exponent of participants divided according to PVS**

| Item | PVS negative  (N=34) | PVS positive  (N=21) | P value |
| --- | --- | --- | --- |
| α | 0.94(0.04) | 0.93(0.05) | 0.871 |
| α1 | 0.94(0.07) | 0.93(0.07) | 0.994 |
| α2 | 0.95(0.10) | 0.92(0.09) | 0.467 |
| △α | -0.01(0.14) | -0.02(0.21) | 0.878 |

PVS: perivascular spaces
