# Supplementary figures and images for: Detecting the fractal physical activity pattern in aged adults with cerebral small vessel disease
Source: Front Aging Neurosci. 2025 Apr 28;17:1569582. doi: 10.3389/fnagi.2025.1569582 (PMC12066675; doi:10.3389/fnagi.2025.1569582)

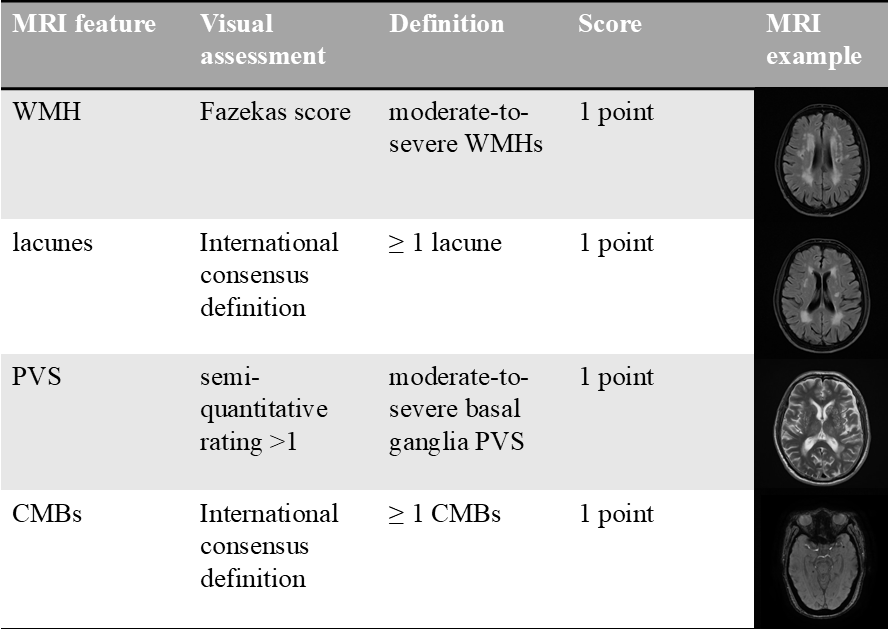

Supplement: Supplementary Figure 1 — Total small-vessel disease score features and categories. [file Image_1.png]
